# AdaFed-LDR: Adaptive Federated Learning with Layerwise Dynamics Regularization for Robust Wi-Fi Localization

**DOI:** 10.3390/s26103148

**Published:** 2026-05-15

**Authors:** Kaito Harada, Hirofumi Natori, Makoto Koike, Hiroshi Mineno

**Affiliations:** 1 Graduate School of Integrated Science and Technology, Shizuoka University, Hamamatsu 432-8011, Shizuoka, Japan; harada.kaito.20@shizuoka.ac.jp; 2 Faculty of Informatics, Shizuoka University, Hamamatsu 432-8011, Shizuoka, Japan; natori.hirofumi.22@shizuoka.ac.jp (H.N.); koike-m@inf.shizuoka.ac.jp (M.K.); 3 College of Informatics, Academic Institute, Shizuoka University, Hamamatsu 432-8011, Shizuoka, Japan; 4 Research Institute of Green Science and Technology, Shizuoka University, Hamamatsu 422-8529, Shizuoka, Japan

**Keywords:** indoor positioning system, Channel State Information (CSI), federated learning, privacy preservation, domain adaptation, non-IID, multipath interference, layerwise dynamics

## Abstract

Wi-Fi Channel State Information (CSI)-based indoor localization enables high-precision positioning, but its deployment across multiple environments faces two major challenges: privacy concerns from centralizing CSI data, and severe statistical heterogeneity (non-IID) arising from the strong environment-dependency of CSI. This heterogeneity creates a stability–plasticity trade-off in federated learning—maintaining precision in known environments (stability) while adapting to unseen domains (plasticity). To address this trade-off, we propose AdaFed-LDR, which combines server-side Confidence-Weighted Adaptive Aggregation with client-side Layerwise Dynamics Regularization (LDR). The aggregation recalibrates client contributions based on feature covariance changes, while LDR imposes depth-dependent constraints—stronger constraints on shallow layers to preserve environment-agnostic features and weaker constraints on deeper layers to allow environment-specific adaptation. Evaluated across 8 indoor environments using Leave-One-Out Cross-Validation and 5 random seeds, AdaFed-LDR achieved a mean localization error (MLE) of 0.41 cm in known environments, corresponding to an 88.2% reduction compared with FedAvg. In domain generalization to unseen environments, AdaFed-LDR achieved an MLE of 218.2±2.8 cm, demonstrating an improvement over FedPos (257.6±14.04 cm). With one adaptation sample per reference point, MLE improved to 21 cm. Ablation experiments confirmed that combining the two proposed components achieved the highest improvement (83.9%) compared with applying them individually, supporting AdaFed-LDR as a reproducible approach to the stability–plasticity trade-off in federated CSI-based localization.

## 1. Introduction

Indoor localization is a foundational technology for smart environments, supporting applications such as IoT device management and robotic navigation [1,2]. While GPS is widely used outdoors, its signals are attenuated inside buildings, resulting in degraded positioning accuracy. Consequently, localization methods that leverage Wi-Fi infrastructure installed in buildings have been investigated [3,4].

Among Wi-Fi-based localization approaches, the use of Channel State Information (CSI) has attracted increasing attention [5,6]. CSI represents the channel characteristics of radio wave propagation between a transmitter and receiver, capturing amplitude and phase variations across multiple frequency bands (subcarriers). The conventional Received Signal Strength Indicator (RSSI) provides only a single aggregate measure of received power, making it difficult to capture fine-grained environmental changes. In contrast, CSI provides channel responses for tens to hundreds of individual subcarriers, enabling the detection of subtle variations in radio propagation caused by changes in receiver position and achieving positioning accuracy on the order of tens of centimeters.

Deep learning approaches have demonstrated competitive accuracy in CSI-based indoor localization [7,8]. DeepFi [7] collects CSI data at multiple reference points (RPs)—measurement locations that serve as position references—within an environment and trains a neural network using the location labels associated with each RP. The trained model estimates the RP to which the receiver is closest based on newly observed CSI patterns. Such methods have achieved high localization accuracy even in multipath environments where radio waves arrive via multiple paths after reflecting off walls and furniture.

To build a practical localization system, it is desirable to train models using data collected from diverse environments. A model trained solely on data from a single building or room tends to overfit to that specific environment, resulting in degraded performance in other settings. Therefore, integrating data from multiple environments with different characteristics—such as office buildings, commercial facilities, and residential spaces—is necessary for training [9,10].

One approach to multi-environment data integration is centralized learning, in which CSI data and location labels collected from all environments are aggregated on a single server for model training. However, centralized learning raises privacy concerns [11]. The combination of CSI data and location labels reveals which CSI patterns are observed at specific positions within an environment. If such information were leaked, it could potentially allow inference of building indoor layouts and obstacle placements, or reconstruction of movement histories of users carrying devices. Additionally, the continuous transmission of high-dimensional CSI data to a server imposes substantial network bandwidth requirements, creating a barrier to large-scale deployment.

Federated Learning (FL) has been explored as an approach to address both privacy preservation and communication efficiency [12]. In FL, each client trains a model locally on its own data without transmitting raw data to an external server. After training, each client sends only its model weights (parameters) to a server. The server aggregates the weights received from multiple clients (e.g., by averaging) to produce a global model, which is then redistributed to each client. By iterating this process, a model trained on data from multiple clients is trained without directly sharing the underlying data, enabling both privacy preservation and communication efficiency.

A key challenge in applying FL to CSI-based localization is distributional heterogeneity across environments. CSI measurements are sensitive to environmental factors, exhibiting different characteristics depending on room geometry, furniture placement, wall materials, and the Wi-Fi hardware used [13,14]. For example, an office environment with abundant metallic equipment and an apartment with predominantly wooden furniture may produce different CSI patterns. This situation, where the statistical properties of data held by each client differ, is referred to as non-Independent and Identically Distributed (non-IID).

FedAvg [12], a representative FL algorithm, constructs the global model by simply averaging local model updates from each client. However, when averaging is performed across non-IID clients, environment-specific features can cancel each other out, resulting in a model that performs inadequately across all environments. Consequently, slow convergence and model instability in the known training environments (source domains) have been reported, along with a tendency toward reduced generalization to new environments (target domains) that did not participate in training [15,16].

Among existing approaches to non-IID data, regularization-based methods such as FedProx [17] and SCAFFOLD [18] constrain each client’s model updates to remain close to the global model. This prevents non-IID clients from updating the model in highly divergent directions and promotes stable convergence in source domains. However, these methods apply constraints of uniform strength across all layers of the model. In deep neural networks, it is known that shallow layers learn generic low-level features (such as edges and textures) while deeper layers learn task-specific high-level features [19]. Applying constraints of identical strength to all layers may result in insufficiently strong constraints on shallow layers—undermining the consistency of generic features that should be shared across environments—while overly constraining deeper layers, potentially hindering appropriate adaptation to each environment [20,21].

Domain adaptation approaches, such as FedPos [22], separate the model into a feature extraction component shared across environments and an environment-specific classification component. The shared component learns environment-independent generic representations, while the specific component is adapted to the characteristics of each environment. This design enables efficient adaptation to a target domain by fine-tuning only the specific component with a small amount of data. However, as the shared component prioritizes generality, it may not fully capture the features necessary for discriminating fine-grained positional differences in source domains. Consequently, while adaptability to target domains may improve, reduced localization precision in source domains has been observed [23,24].

In this way, existing methods tend to prioritize either stable operation in source domains (stability) or deployment to target domains (plasticity), making it difficult to achieve both at a high level simultaneously [25].

A practical localization system requires from both stability and plasticity. For example, when deploying robots with localization capability across multiple office buildings, it is desirable to maintain high-precision positioning in currently operational buildings while enabling adaptation to new buildings using only a small number of RP measurements. Since collecting large amounts of training data for each target domain is often impractical, zero-shot and few-shot performance are important considerations.

To address the stability–plasticity trade-off in federated CSI-based localization, this paper proposes AdaFed-LDR, which combines server-side Confidence-Weighted Adaptive Aggregation (hereafter referred to as adaptive aggregation) with client-side Layerwise Dynamics Regularization (LDR).

The adaptive aggregation mechanism weights each client update according to its estimated reliability. Specifically, it monitors the feature covariance matrices extracted by each client and assigns higher reliability to clients that learn temporally stable features, while suppressing the influence of unstable updates affected by noise. This aims to mitigate the adverse effects of excessive variability in client updates on the global model.

Client-side LDR applies regularization of different strengths to each layer of the network. Shallow layers, which extract generic low-level features such as edges and textures that are less dependent on the specific environment, receive stronger regularization to maintain consistency with the global model. Deeper layers, which learn task- and environment-specific high-level features, receive weaker regularization to permit adaptation to each environment’s data. Through this differentiation, LDR aims to preserve the consistency of generic features that should be shared across environments while also allowing the learning of features necessary for position discrimination within each environment.

By combining both mechanisms, AdaFed-LDR aims to achieve both high precision in source domains and effective adaptation to target domains.

To validate the proposed method, we conducted experiments using CSI datasets collected from 8 diverse environments. We adopted Leave-One-Out Cross-Validation (LOOCV), designating 7 of the 8 environments as source domains participating in training and the remaining 1 as a target domain excluded from training, evaluating all 8 possible configurations. Statistical tests using 5 different random seeds were performed to assess the significance of differences between methods.

In the experiments, stability was evaluated based on localization accuracy in source domains. Plasticity was assessed based on zero-shot performance in target domains (measuring generalization) and few-shot performance (measuring adaptation capability). Representative FL methods, including FedAvg and FedProx, were used as comparison baselines.

The main contributions of this paper are as follows:

- In federated CSI-based localization, this study proposes AdaFed-LDR to address the severe stability–plasticity trade-off caused by environmental multipath effects. Its fundamental novelty lies in a complementary architecture specifically designed for the physical characteristics of RF signals, tightly coupling local adaptation with representation-space reliability estimation during global aggregation.
- Through evaluation on source domains, this study shows that AdaFed-LDR achieves higher localization accuracy compared to existing FL methods, with smaller performance variance across different random seeds. These results suggest that the proposed method contributes to stable operation in source domains.
- Through evaluation on target domains, this study confirms that AdaFed-LDR mitigates the severe performance degradation observed in existing methods. In the few-shot setting, while adaptation accuracy may be lower than that of domain adaptation methods in some cases, the performance variance remains small, indicating that the design goal of balancing stability and plasticity is met.
- Through ablation experiments, this study confirms that both adaptive aggregation and LDR contribute to performance improvement, and that combining the two components achieves the highest overall accuracy and lowest variance compared to their individual applications, validating their complementary integration.

## 2. Related Work

### 2.1. Wi-Fi CSI-Based Indoor Localization and Deep Learning

The sensing modality for Wi-Fi-based indoor localization has shifted from RSSI to CSI. While RSSI is straightforward to implement, it provides only a single aggregate measure of received power, limiting positioning accuracy to the order of several meters [3,4]. In contrast, CSI captures amplitude and phase information for each subcarrier in Orthogonal Frequency Division Multiplexing (OFDM) communication, reflecting fine-grained variations in radio propagation that RSSI cannot detect [8]. This granularity has made CSI an attractive sensing medium for high-accuracy localization.

The dominant approach to CSI-based indoor localization has shifted from model-based methods to fingerprinting. Model-based methods extract Angle of Arrival (AoA) or Time of Flight (ToF) from CSI and determine the position via triangulation or trilateration [26,27]. SpotFi [26] is a representative example, achieving high-accuracy AoA/ToF estimation using super-resolution algorithms. However, model-based methods rely on the assumption that a Line-of-Sight (LoS) path exists between transmitter and receiver. Since AoA and ToF estimation depend on direct-path characteristics, accuracy degrades substantially in Non-Line-of-Sight (NLoS) environments—common in practice due to occlusion by walls and furniture—where the direct path is attenuated or absent [28,29]. Fingerprinting methods construct a database of CSI measurements taken at each RP within an environment and estimate position by matching new measurements against this database [30]. Because this approach does not rely solely on the direct path and instead learns the overall CSI pattern—including reflections and scattering—as a location-specific signature, it achieves relatively robust performance even in NLoS environments.

With the adoption of fingerprinting, CSI-based localization methods have evolved from classical machine learning to deep learning [7,31]. Early fingerprinting approaches employed k-nearest neighbors or support vector machines; however, CSI data exhibit high-dimensional and nonlinear characteristics across tens to hundreds of subcarriers, which limited the representational capacity of these models. DeepFi [7] addressed this limitation by using deep autoencoders to automatically extract CSI features, overcoming the constraints of manual feature engineering. DLoc [31] introduced convolutional neural networks (CNNs), treating CSI data as two-dimensional images to effectively capture spatial correlations across subcarriers and antennas. These methods have enabled positioning accuracy on the order of tens of centimeters.

However, these deep learning-based methods assume centralized learning, where data collected from a single or a few environments is aggregated on a central server, leaving several practical challenges unresolved. First, the combination of CSI data and location labels may enable the inference of building interior layouts or reconstruction of user movement histories, raising privacy concerns [11,32]. Second, continuous transmission of high-dimensional CSI data to a server imposes substantial bandwidth requirements, posing scalability challenges for large-scale deployment [33]. Third, because CSI characteristics are strongly environment-dependent, domain shift—where the statistical distributions of training and evaluation data differ—readily occurs, and models trained in limited environments tend to exhibit degraded accuracy in other settings [13,14]. To address these issues, the application of federated learning, which enables distributed model training without uploading raw data to a server, has been explored.

### 2.2. Federated Learning Under Non-IID Distributions

Federated learning is a framework in which multiple clients participate in model training without sharing their local data, aggregating learned knowledge into a global model [12]. This mechanism supports privacy preservation and reduces communication costs. Moreover, when multiple clients distributed across diverse environments participate in training, the learning of generic representations that are not overly dependent on any specific environment is promoted, which may improve resilience to domain shift.

However, the non-IID nature of data poses a serious challenge when applying FL to CSI-based indoor localization [15,16]. Because CSI exhibits different characteristics depending on room geometry, furniture placement, and wall materials, the statistical properties of data held by each client vary considerably across environments. FedAvg, a standard FL algorithm, constructs the global model by simply averaging client model updates; under such non-IID conditions, convergence tends to become unstable and accuracy may degrade.

#### 2.2.1. Regularization-Based Approaches

One approach to addressing non-IID data is to constrain local model updates from diverging too far from the global model through regularization. FedProx [17] adds a proximal term based on the distance from the global model to the local loss function, suppressing client drift. SCAFFOLD [18] tracks the gradient difference between the client and the server as a control variate, correcting the update direction to achieve smoother convergence.

From a representation learning perspective, contrastive learning-based methods have also been proposed. MOON [34] introduces a contrastive loss that encourages each client’s feature representations to be close to that of the global model while being distant from those of the previous local model. FedProc [35] promotes the learning of a consistent feature space across clients through contrastive learning using class prototypes.

However, these methods face limitations in the context of CSI-based indoor localization. Regularization-based methods apply constraints of uniform strength across all layers of the network. Since different layers in deep neural networks serve distinct roles, uniform constraints may be inappropriate [36,37]. Regarding contrastive learning-based methods, CSI exhibits physical continuity—producing similar patterns at spatially adjacent RPs—which may conflict with the design principle of contrastive learning that aims to clearly separate the feature representations of different classes (RPs).

#### 2.2.2. Domain Adaptation and Personalization Approaches

An alternative approach is to make parts of the model client-specific [22,38,39] or assuming fine-tuning in new environments [40,41]. FedPos [22], which integrates transfer learning, separates the model into components shared across environments and environment-specific components, improving adaptability to new environments. Per-FedAvg [40] introduces the concept of Model-Agnostic Meta-Learning (MAML) into FL, aiming to learn a global model that can be efficiently fine-tuned with a small amount of data.

However, these methods tend to sacrifice stability by prioritizing plasticity [23,24].

### 2.3. Layerwise Adaptation and Dynamic Aggregation

As noted in the previous subsection, Yosinski et al. [42] demonstrated that different layers of deep neural networks learn features of different natures. By analyzing networks trained on ImageNet [43], they showed that shallow layers learn generic features transferable across datasets, while deeper layers learn task-specific features. This finding motivates layer-specific strategies in federated learning.

Several methods apply layer-specific processing in FL, including approaches that share only shallow layers while keeping deeper layers local [44,45], and methods that apply different learning rates to different layers [46]. However, these methods rely on predefined fixed rules and have limited capacity to adjust dynamically in response to training progress or environment complexity.

Methods that vary aggregation weights across clients have also been proposed, including data-quantity-based weighting [47] and approaches based on loss values or gradient similarity [48].

### 2.4. Positioning of This Work

The analysis of related work presented above indicates that achieving both stability and plasticity is important when applying FL to CSI-based indoor localization. Existing methods tend to prioritize either stability, as in regularization-based approaches, or plasticity, as in personalization-based approaches. Although individual techniques such as layerwise regularization and covariance-based adaptation have been explored in the broader machine learning literature, the fundamental novelty of AdaFed-LDR lies in its problem-driven and complementary integration of these components, explicitly tailored to the physical characteristics of Wi-Fi CSI sensing.

The proposed AdaFed-LDR aims to address this stability–plasticity trade-off. LDR applies stronger constraints to shallow layers to maintain the consistency of generic features that should be shared across environments, while applying weaker constraints to deeper layers to permit adaptation to each environment’s data. Furthermore, inspired by CORAL [49,50]—which demonstrated that feature covariance matrices are effective indicators for capturing domain-specific statistical properties—we introduce adaptive aggregation based on changes in feature covariance matrices. Specifically, clients whose covariance matrices exhibit large changes before and after local training are considered potentially affected by environmental noise or local overfitting, and their aggregation weights are reduced to suppress the influence of unreliable updates on the global model. The combination of dynamic regularization that accounts for layer-specific roles and aggregation based on feature-space consistency represents a distinctive aspect of this work.

## 3. Methodology

**Figure 1:**
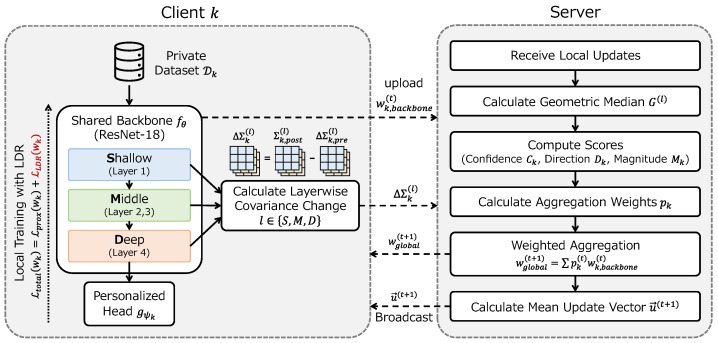
Overview of the AdaFed-LDR framework. Each client computes feature covariance matrices before and after local training with Layerwise Dynamics Regularization (LDR), and transmits the backbone parameters along with covariance changes to the server. The server computes confidence, direction, and magnitude scores for each client based on these covariance changes, derives aggregation coefficients using EMA-smoothed softmax, and updates the global backbone accordingly.

In this section, we present AdaFed-LDR. The proposed framework consists of (1) server-side adaptive aggregation and (2) client-side Layerwise Dynamics Regularization (LDR). Figure 1 provides an overview of the framework, and the overall procedure is summarized in Algorithm 1.
**Algorithm 1** AdaFed-LDR**Require:** Number of clients *K*, global rounds *R*, local epochs *E*.**Ensure:** Trained global backbone $w_{\mathrm{backbone}}^{\left(R\right)}$, personalized heads ${\left\{g_{\psi_{k}}\right\}}_{k=1}^{K}$. 1: Initialize global model $w^{\left(0\right)}$, average update vector ${\bar{u}}^{\left(0\right)}\leftarrow 0$, EMA scores ${\tilde{s}}_{k}^{\left(0\right)}\leftarrow 0$. 2: **for** round t=0 to R−1 **do** 3:    Broadcast $w_{\mathrm{backbone}}^{\left(t\right)}$ and ${\bar{u}}^{\left(t\right)}$ to all clients. 4:    **for** each client k∈{1,…,K} in parallel **do** 5:        Compute pre-training covariance $\Sigma_{k,\mathrm{pre}}^{\left(l\right)}$ for each level *l*. 6:        Perform *E* epochs of local training (minimize $L_{\mathrm{total}}$ in Equation (16)). 7:        Compute post-training covariance $\Sigma_{k,\mathrm{post}}^{\left(l\right)}$ for each level *l*. 8:        Send $w_{k,\mathrm{backbone}}$ and $\left\{\Delta \Sigma_{k}^{\left(l\right)}\right\}$ to the server. 9:    **end for**10:    **// Server-side aggregation**11:    **for** each level l∈{S,M,D} **do**12:        Compute geometric median $G^{\left(l\right)}$ (Equation (6)).13:    **end for**14:    **for** each client *k* **do**15:        Compute confidence $C_{k}^{\left(l\right)}$, direction $D_{k}^{\left(l\right)}$, magnitude $M_{k}^{\left(l\right)}$.16:        Contribution score: $s_{k}^{\left(t\right)}\leftarrow \sum_{l}\alpha_{l}\cdot C_{k}^{\left(l\right)}\cdot D_{k}^{\left(l\right)}\cdot M_{k}^{\left(l\right)}$.17:        Apply EMA: ${\tilde{s}}_{k}^{\left(t\right)}\leftarrow \beta {\tilde{s}}_{k}^{\left(t-1\right)}+\left(1-\beta \right)s_{k}^{\left(t\right)}$.18:    **end for**19:    Compute aggregation coefficients: $p_{k}^{\left(t\right)}\leftarrow \frac{\exp({\tilde{s}}_{k}^{\left(t\right)}/T)}{\sum_{j=1}^{K}\exp\left({\tilde{s}}_{j}^{\left(t\right)}/T\right)}$.20:    $w_{\mathrm{backbone}}^{\left(t+1\right)}\leftarrow \sum_{k=1}^{K}p_{k}^{\left(t\right)}w_{k,\mathrm{backbone}}$.21:    Update average update vector ${\bar{u}}^{\left(t+1\right)}$ (Equation (14)).22: **end for**23: **return** $w_{\mathrm{backbone}}^{\left(R\right)}$, ${\left\{g_{\psi_{k}}\right\}}_{k=1}^{K}$.

### 3.1. Problem Formulation

We consider a federated learning setting with *K* clients (environments). The *k*-th client holds a private dataset $D_{k}={\left\{\left(x_{i},y_{i}\right)\right\}}_{i=1}^{N_{k}}$, where $x_{i}$ denotes CSI data and $y_{i}$ denotes the corresponding location label. We formulate the indoor localization task as a classification problem over discrete spatial reference points (RPs).

#### 3.1.1. Adaptive Grid Converter

The arrangement of reference points varies across environments. To handle this heterogeneity, we introduce an Adaptive Grid Converter that constructs a unified label space. Let $U_{k}={\left\{\left(x_{j},y_{j}\right)\right\}}_{j=1}^{M_{k}}$ be the set of unique ground-truth coordinates in the dataset of client *k*. The global reference point set is constructed as:

(1) $$U_{\mathrm{global}}=\overset{K}{\underset{k=1}{⋃}}U_{k}.$$

The converter assigns a unique class ID $c_{j}\in \left\{0,1,\ldots ,\right|U_{\mathrm{global}}\left|-1\right\}$ to each coordinate tuple $\left(x_{j},y_{j}\right)$ based on lexicographical sorting (first by *x*-coordinate, then by *y*-coordinate).

#### 3.1.2. Global Objective Function

The objective is to minimize the global loss function:

(2) $$\min_{w}L\left(w\right)=\sum_{k=1}^{K}p_{k}L_{k}\left(w\right),$$

where $L_{k}\left(w\right)$ is the label-smoothing cross-entropy loss of client *k*, and $p_{k}$ is the adaptive aggregation coefficient computed by the proposed method.

### 3.2. Network Architecture

We adopt ResNet-18 [51], pre-trained on ImageNet, as the backbone network. The model consists of two components:

- Shared Backbone $f_{\theta }$: The convolutional layers of ResNet-18, including the initial stem (conv1, bn1, relu, maxpool), four residual blocks (layer1–layer4), and global average pooling.
- Local Classification Head $g_{\psi_{k}}$: A fully connected layer that maps the 512-dimensional feature vector to $|U_{\mathrm{global}}|$ classes.

The shared backbone is aggregated across clients via adaptive aggregation (Algorithm 1, line 20). Each client maintains a personalized local classification head to handle environment-specific variations.

### 3.3. Layerwise Feature Extraction and Covariance Analysis

A central component of the proposed method is the analysis of feature changes at multiple depths of the network. To assess the consistency of each client’s learning, we compute feature covariance matrices at three hierarchical levels of the ResNet-18 backbone:

- Shallow Level *S*: Output of layer1 (64 channels: $d_{S}=64$).
- Mid Level *M*: Output of layer3 (256 channels: $d_{M}=256$).
- Deep Level *D*: Output of layer4 after global average pooling (512 channels: $d_{D}=512$).

#### 3.3.1. Feature Extraction Procedure

For each level l∈{S,M,D}, features are extracted from all training samples in client *k*’s dataset. Let $h_{i}^{\left(l\right)}\in R^{d_{l}}$ denote the feature vector of sample *i* at level *l*, obtained by applying global average pooling to the spatial feature map followed by flattening. The feature matrix of client *k* at level *l* is:

(3) $$H_{k}^{\left(l\right)}={\left[h_{1}^{\left(l\right)},h_{2}^{\left(l\right)},\ldots ,h_{N_{k}}^{\left(l\right)}\right]}^{⊤}\in R^{N_{k}\times d_{l}}.$$

#### 3.3.2. Covariance Matrix Computation

The covariance matrix captures the correlation structure among features. Tracking how this structure changes during local training enables the assessment of each client’s learning consistency. The sample covariance matrix of client *k* at level *l* is computed as:

(4) $$\Sigma_{k}^{\left(l\right)}=\frac{1}{N_{k}-1}{\left(H_{k}^{\left(l\right)}-{\bar{H}}_{k}^{\left(l\right)}\right)}^{⊤}\left(H_{k}^{\left(l\right)}-{\bar{H}}_{k}^{\left(l\right)}\right),$$

where ${\bar{H}}_{k}^{\left(l\right)}=\frac{1}{N_{k}}\sum_{i=1}^{N_{k}}h_{i}^{\left(l\right)}$ is the mean feature vector. This covariance computation is performed both before and after local training (Algorithm 1, lines 5 and 7).

### 3.4. Server-Side: Adaptive Aggregation

The server computes aggregation coefficients based on the reliability of each client’s update. The goal is to suppress the influence of unreliable updates—those affected by noise or deviating substantially from the collective trend—thereby constructing a more robust global model.

We first describe the computation of covariance changes and the geometric median, then formulate the confidence, direction, and magnitude scores, and finally present the integrated aggregation coefficient computation and global model update procedure. These operations correspond to lines 11–20 in Algorithm 1.

#### 3.4.1. Covariance Change and Geometric Median

For each level *l*, client *k* computes the change in covariance before and after local training:

(5) $$\Delta \Sigma_{k}^{\left(l\right)}=\Sigma_{k,\mathrm{post}}^{\left(l\right)}-\Sigma_{k,\mathrm{pre}}^{\left(l\right)},$$

where $\Sigma_{k,\mathrm{pre}}^{\left(l\right)}$ and $\Sigma_{k,\mathrm{post}}^{\left(l\right)}$ are the covariance matrices before and after local training, respectively.

The server aggregates these covariance changes and computes the geometric median $G^{\left(l\right)}$, which represents the typical trend of changes across all clients (Algorithm 1, line 12). Unlike the arithmetic mean, the geometric median is less sensitive to outliers [52]:

(6) $$G^{\left(l\right)}=\arg\min_{G}\sum_{k=1}^{K}N_{k}{∥G-\Delta \Sigma_{k}^{\left(l\right)}∥}_{F},$$

where ${∥\cdot ∥}_{F}$ denotes the Frobenius norm. The geometric median is computed iteratively using the Weiszfeld algorithm [52].

#### 3.4.2. Confidence Score Formulation

To evaluate the reliability of client *k*’s update at level *l*, we define a confidence score $C_{k}^{\left(l\right)}\in \left[0,1\right]$. This score assesses the quality of an update from three perspectives:

(7) $$C_{k}^{\left(l\right)}=\sigma \left(-\lambda_{1}\mathrm{Var}\left(\Delta \Sigma_{k}^{\left(l\right)}\right)-\lambda_{2}\frac{∥\Delta \Sigma_{k}^{\left(l\right)}{∥}_{F}}{∥\Sigma_{k,\mathrm{pre}}^{\left(l\right)}{∥}_{F}+\epsilon }+\lambda_{3}\log\left(N_{k}+1\right)\right),$$

where σ(·) is the sigmoid function that normalizes the score to [0,1]; $\lambda_{1},\lambda_{2},\lambda_{3}$ are hyperparameters controlling the influence of each term; and ϵ is a small constant ($10^{-5}$) to prevent division by zero.

The design rationale of each term is as follows:

##### First Term: Uniformity of Change

$\mathrm{Var}(\Delta \Sigma_{k}^{\left(l\right)})$ is the element-wise variance of the covariance change matrix, measuring whether changes in inter-feature correlations are uniform. In consistent learning, many inter-feature correlations change cooperatively, resulting in relatively uniform element-wise changes. In contrast, learning affected by noise or local overfitting tends to produce non-uniform patterns where only specific feature-pair correlations change abruptly while others remain static. This term penalizes such biased changes.

##### Second Term: Total Magnitude of Change

$∥\Delta \Sigma_{k}^{\left(l\right)}{∥}_{F}$ measures the total magnitude of covariance change. While the first term evaluates the dispersion of changes, the second term evaluates their overall scale. Even if changes are uniform (i.e., the first term is small), a large overall shift in the feature correlation structure during a single round of local training may indicate that the client’s data distribution differs substantially from those of other clients. Strongly reflecting such updates in the global model could negatively affect other clients; therefore, large changes are penalized.

##### Third Term: Statistical Reliability

Clients with more samples $N_{k}$ produce more reliable covariance matrix estimates. The logarithmic scale ensures moderate adjustment even when sample sizes vary by large factors.

#### 3.4.3. Direction Score and Magnitude Score

In addition to the confidence score, we evaluate the extent to which each client’s update aligns with the overall trend.

The direction score measures the cosine similarity between client *k*’s covariance change and the geometric median (the typical change direction across all clients):

(8) $$D_{k}^{\left(l\right)}=\max\left(0,\frac{\mathrm{vec}{\left(\Delta \Sigma_{k}^{\left(l\right)}\right)}^{⊤}\mathrm{vec}\left(G^{\left(l\right)}\right)}{∥\mathrm{vec}\left(\Delta \Sigma_{k}^{\left(l\right)}\right){∥}_{2}{∥\mathrm{vec}\left(G^{\left(l\right)}\right)∥}_{2}}\right),$$

where vec(·) denotes the operation of flattening a matrix into a vector. The cosine similarity ranges from −1 to 1; negative values (indicating updates in the opposite direction) are clipped to zero, as such updates may hinder convergence of the global model.

The magnitude score captures the scale of the update:

(9) $$M_{k}^{\left(l\right)}={∥\Delta \Sigma_{k}^{\left(l\right)}∥}_{F}.$$

#### 3.4.4. Aggregation Coefficient Computation

The contribution score $s_{k}^{\left(t\right)}$ of client *k* integrates information across all levels using layer importance weights $\alpha_{l}$ (Algorithm 1, line 16):

(10) $$s_{k}^{\left(t\right)}=\sum_{l\in \{S,M,D\}}\alpha_{l}\cdot C_{k}^{\left(l\right)}\cdot D_{k}^{\left(l\right)}\cdot M_{k}^{\left(l\right)},$$

where $\alpha_{S},\alpha_{M},\alpha_{D}$ are hyperparameters reflecting the importance of each level. Since deeper-layer features exhibit higher environment dependence, assigning larger importance to the deep level enables more sensitive detection of inter-environment differences.

To smooth the contribution scores and stabilize training, Exponential Moving Average (EMA) smoothing is applied (Algorithm 1, line 17):

(11) $${\tilde{s}}_{k}^{\left(t\right)}=\beta {\tilde{s}}_{k}^{\left(t-1\right)}+\left(1-\beta \right)s_{k}^{\left(t\right)},$$

where β∈[0,1] is the EMA coefficient.

The final aggregation coefficients are computed via softmax normalization with temperature *T* (Algorithm 1, line 19):

(12) $$p_{k}^{\left(t\right)}=\frac{\exp({\tilde{s}}_{k}^{\left(t\right)}/T)}{\sum_{j=1}^{K}\exp\left({\tilde{s}}_{j}^{\left(t\right)}/T\right)}.$$

#### 3.4.5. Global Model Update

The global backbone parameters are updated as a weighted average using the aggregation coefficients (Algorithm 1, line 20):

(13) $$w_{\mathrm{backbone}}^{\left(t+1\right)}=\sum_{k=1}^{K}p_{k}^{\left(t\right)}w_{k,\mathrm{backbone}}^{\left(t\right)},$$

where $w_{k,\mathrm{backbone}}^{\left(t\right)}$ denotes the backbone parameters of client *k* after local training.

### 3.5. Client-Side: Layerwise Dynamics Regularization (LDR)

LDR exploits the property that different network layers serve distinct roles. Shallow layers tend to extract generic features (e.g., edges, textures) that are less dependent on specific environments, while deeper layers learn higher-level features that may be more environment-specific [42]. LDR applies constraints of varying strength according to this hierarchical structure, aiming to maintain consistency of generic features in shallow layers while permitting adaptation to each environment in deeper layers.

We first describe the layer grouping and the role of each group, then define the average update vector and formulate the LDR loss function, and finally present the overall local training objective. The local training procedure corresponds to line 6 in Algorithm 1.

#### 3.5.1. Layer Grouping

The ResNet-18 backbone is divided into three groups with different regularization strengths:

- Shallow Group ($\eta_{S}$): Includes the initial stem (conv1, bn1, relu, maxpool) and layer1.
- Mid Group ($\eta_{M}$): Includes layer2 and layer3.
- Deep Group ($\eta_{D}$): Includes layer4.

The local classification head (fully connected layer) is excluded from LDR and is trained independently on each client.

#### 3.5.2. Average Update Vector

The server records the average parameter update direction across all clients from the previous round (Algorithm 1, line 21):

(14) $${\bar{u}}^{\left(t-1\right)}=\frac{1}{K}\sum_{k=1}^{K}\left(w_{k}^{\left(t-1\right)}-w^{\left(t-1\right)}\right).$$

This average update vector represents the mean direction in which all clients updated the model in the previous round. By constraining each client’s local update to avoid large deviations from this direction, it aims to maintain consistency of update directions across clients. The average update vector is broadcast to all clients together with the global model at the beginning of each round (Algorithm 1, line 3).

#### 3.5.3. LDR Loss Function

The LDR loss encourages each client’s update direction to align with the average update vector:

(15) $$L_{\mathrm{LDR}}\left(w_{k}\right)=\frac{\lambda_{\mathrm{LDR}}}{2}\sum_{l\in \{S,M,D\}}\eta_{l}\sum_{\theta \in \Theta_{l}}{∥\left(w_{k}^{\left(\theta \right)}-w_{\mathrm{global}}^{\left(\theta \right)}\right)-{\bar{u}}^{\left(t-1,\theta \right)}∥}_{2}^{2},$$

where:

- $\Theta_{l}$ is the set of parameters belonging to layer group *l*.
- $w_{k}^{\left(\theta \right)}$ is the current local parameter of client *k*, which is updated during local training.
- $w_{\mathrm{global}}^{\left(\theta \right)}$ is the global parameter received from the server at the beginning of the round, which remains fixed during local training.
- ${\bar{u}}^{\left(t-1,\theta \right)}$ is the corresponding component of the average update vector.
- $\lambda_{\mathrm{LDR}}$ is the overall coefficient of the LDR loss.

This loss penalizes the degree to which the current update direction $\left(w_{k}^{\left(\theta \right)}-w_{\mathrm{global}}^{\left(\theta \right)}\right)$ deviates from the previous round’s average update direction ${\bar{u}}^{\left(t-1,\theta \right)}$.

#### 3.5.4. Local Training Objective

Each client *k* minimizes the following composite objective during local training:

(16) $$L_{\mathrm{total}}\left(w_{k}\right)=L_{\mathrm{task}}\left(w_{k};D_{k}\right)+\frac{\mu }{2}{∥w_{k}-w_{\mathrm{global}}∥}_{2}^{2}+L_{\mathrm{LDR}}\left(w_{k}\right),$$

where:

- $L_{\mathrm{task}}$ is the label-smoothing cross-entropy loss for the classification task.
- The second term is the proximal regularization from FedProx [17], which prevents rapid divergence from the global model.

## 4. Experimental Setup

### 4.1. Dataset

We collected Wi-Fi CSI data across 8 indoor environments, including a server room and a residential apartment. These environments differ in room geometry, furniture placement, and wall materials, constituting a non-IID setting where the statistical properties of CSI vary across environments. Data collection was conducted from April to October 2025.

#### 4.1.1. Hardware Configuration

In this experiment, a laptop (DELL GX83/MLE) transmitted ICMP echo request packets to a Wi-Fi access point (ASUS RT-AC86U) at a rate of 800 packets per second. Each ICMP echo request packet, generated using the standard Windows Command Prompt, contained a 32-byte payload and resulted in a 60-byte IP packet. The CSI data corresponding to each transmitted packet were captured by passively intercepting the over-the-air transmissions using a Raspberry Pi 4B running the Nexmon CSI tool in monitor mode. In the Nexmon CSI tool, IP filtering was used to capture only the packets exchanged between the laptop and the access point. Communication was conducted in the 5.0 GHz band with a channel bandwidth of 80 MHz, corresponding to 256 OFDM subcarriers under the Wi-Fi specification. The choice of channel bandwidth affects the number of available subcarriers. To obtain richer spatial information, we selected 80 MHz. Accordingly, up to 800 CSI samples, each containing 256 subcarrier values, could be obtained per second.

#### 4.1.2. Reference Point Layout and Data Collection Procedure

**Figure 2:**
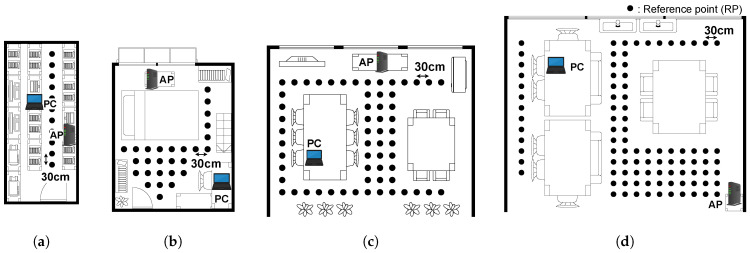
Floor plans of four representative environments. Dots indicate reference points at 30 cm spacing. All environments use 5 GHz Wi-Fi with Raspberry Pi 4B receivers. (**a**) Env A: Server Room (Info. Bldg. 1, 4F). (**b**) Env B: Residential Apartment. (**c**) Env D: Common Area (Info. Bldg. 1, 1F). (**d**) Env H: Common Area (Eng. Bldg. 6, 1F).

Reference points (RPs) were arranged in a grid with 30 cm spacing in each environment. Figure 2 shows the floor plans of four representative environments, where dots indicate RP locations. Table 1 summarizes all environments. Environments A and B are small-scale spaces, while Environments C–H are common areas within a university campus.

At each RP, a single receiver was sequentially moved and placed directly on the floor, and CSI data were collected for one minute. By placing the device directly on the floor rather than using a tripod or having a person hold it, we avoided introducing unpredictable multipath interference or signal absorption that such objects or human bodies would cause. Furthermore, this study explicitly assumes a static environment with no moving objects within the localization area. Therefore, during collection, other people were kept out of the measurement area to ensure the captured CSI reflected only the static channel characteristics of the environment.

#### 4.1.3. Data Splitting

We employed Leave-One-Out Cross-Validation (LOOCV) across the 8 environments, designating 7 as source domains and the remaining 1 as the target domain. This design enables measurement of generalization performance to environments not observed during training.

Chronological splitting was adopted for both source and target domains. For source domains, each environment’s data were treated as an individual dataset, with the first 80% in chronological order used for training/validation and the remaining 20% for testing. The training/validation portion was further split 8:2 into training and validation sets. For the target domain, a fixed number of initial samples were reserved for fine-tuning, and the remainder was used for testing. This splitting prevents temporal leakage—the scenario where a model is trained on temporally later data and tested on earlier data.

Performance was evaluated in a few-shot setting where only *K* samples per RP were used for model fine-tuning, with K∈{0,1,5}. Specifically, “Zero-shot” (*K* = 0) evaluates pure generalization by applying the source-domain-trained global model directly to the target domain without any adaptation. The “1-shot” and “5-shot” settings evaluate the model’s adaptation capability using a strictly limited calibration budget. Given the 800 Hz transmission rate and a window width of 250 packets, one sample corresponds to approximately 0.31 s. Thus, “1-shot” (*K* = 1) indicates that the model is fine-tuned using an additional 0.31 s of CSI data per RP, while “5-shot” (*K* = 5) utilizes approximately 1.56 s of data per RP. The test set consisted of all samples excluding the chronologically first $2\times K_{\max}=10$ samples. During adaptation, the first *K* samples per RP were used for training and the subsequent *K* samples for validation.

### 4.2. Preprocessing

**Figure 3:**
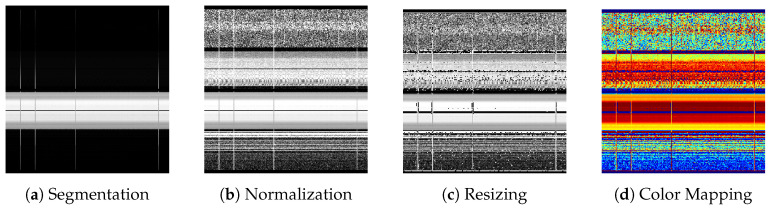
Output images at each stage of the preprocessing pipeline. Sliding window (width = 250, stride = 125) extracts temporal segments, robust group normalization suppresses outliers while preserving band-specific features, and JET colormap conversion produces 3-channel input for the ImageNet-pretrained backbone.

The pipeline for converting CSI time-series data into the input format for the ResNet-18 backbone consists of the following four stages (Figure 3):

1. Segmentation: The time-series CSI data (256 subcarriers) was segmented using a sliding window with width W=250 packets and stride S=125 packets (50% overlap), yielding a 256×250 matrix where the vertical axis corresponds to subcarriers and the horizontal axis to time.
2. Normalization: The 256 subcarriers were divided into G=4 groups (64 subcarriers each), and robust normalization based on the 10th and 90th percentile amplitudes was applied within each group. Normalizing all 256 subcarriers simultaneously risks suppressing lower-amplitude bands when certain bands dominate. Group-wise normalization mitigates this by preserving band-specific characteristics.
3. Resizing: The normalized matrix was resized to 224×224 pixels using bicubic interpolation to match the input size of the ImageNet-pretrained ResNet-18. Interpolation was deliberately adopted rather than simply discarding 32 subcarriers to prevent the potential loss of highly informative, frequency-selective effective subcarriers that capture distinct multipath characteristics.
4. Color Mapping: The single-channel grayscale image was converted to a 3-channel RGB image using the JET colormap. Since the pretrained model was trained on RGB images, this conversion is expected to be more effective for transfer learning than simply replicating the grayscale channel across 3 channels.

### 4.3. Implementation Details

#### 4.3.1. Model Configuration

We employed ResNet-18 [51], initialized with ImageNet [43] pretrained weights, as the backbone. As described in Section 3.2, the model consists of a shared backbone $f_{\theta }$ and a local classification head $g_{\psi_{k}}$. The local classification head is a fully connected layer that takes a 512-dimensional feature vector as input and has an output dimension corresponding to the number of RPs in each environment.

#### 4.3.2. Federated Learning Phase

In the federated learning phase, 7 source domains served as clients in a full-participation setting where all clients participated in each communication round. We used the AdamW optimizer (learning rate 0.001, weight decay 0.01) with a StepLR scheduler (step size 50 rounds, decay rate γ=0.5) and a batch size of 512. The global model was trained for 300 communication rounds, with each client performing 1 epoch of local training per round before server-side aggregation. The model from the round with the best validation performance was saved.

#### 4.3.3. Few-Shot Adaptation Phase

The learning rate was reduced to 0.0001, and training was conducted for 80 epochs. After all epochs, the model from the epoch with the best validation performance was selected as the final model.

#### 4.3.4. Hyperparameters

Table 2 lists the main hyperparameters of AdaFed-LDR. Hyperparameters for the federated learning and adaptation phases were set with reference to values reported in the original papers of FedAvg and FedPos. For the AdaFed-LDR-specific parameters, we did not conduct exploratory hyperparameter tuning to search for fold-specific or performance-maximizing settings; instead, fixed values were used consistently across all LOOCV folds and all random seeds.

The aggregation temperature was set to T=1.0 to preserve differences in contribution scores while avoiding excessively peaked client weights. The confidence coefficients $\lambda_{1},\lambda_{2},\lambda_{3}$ were set so that the three terms in the confidence score—variance of covariance change, normalized Frobenius norm, and logarithmic sample size—contribute at comparable numerical scales, rather than allowing any single term to dominate the score. These three terms were intended to balance complementary aspects of update reliability: irregularity of covariance change, overall magnitude of feature-space drift, and statistical reliability of covariance estimation.

The choice of three feature levels for covariance tracking was guided by the hierarchical structure of ResNet-18. Specifically, we used the outputs of layer1, layer3, and layer4 as representative shallow, intermediate, and deep feature levels, respectively. This design was intended to capture coarse changes in feature dynamics across the network while keeping computational and communication overhead manageable.

The LDR regularization coefficient was set to $\lambda_{\mathrm{LDR}}=0.001$ so that regularization acts as a soft constraint without preventing local learning. The layer-specific regularization coefficients $\eta_{S}$, $\eta_{M}$, $\eta_{D}$ were configured to impose stronger constraints on shallower layers and weaker constraints on deeper layers, following the transfer-learning interpretation that shallow layers tend to encode more generic features, whereas deeper layers capture more environment-specific representations [42]. Consistent with the same rationale, the layer importance weights $\alpha_{S}$, $\alpha_{M}$, $\alpha_{D}$ were assigned as monotonically increasing values (0.25, 0.35, 0.40) to place slightly greater emphasis on deeper feature levels during adaptive aggregation.

The EMA coefficient β=0.9 was set to balance the retention of historical statistics with responsiveness to new updates. The proximal regularization coefficient μ=0.01 follows the value used in FedProx to prevent excessive divergence from the global model.

#### 4.3.5. Computing Environment

The implementation used Python 3.13 and PyTorch 2.9 (Torchvision 0.24), executed with CUDA 13.0 on an NVIDIA RTX 6000 Ada Generation GPU (48 GB VRAM). We explicitly note that this high-performance GPU was used exclusively as an offline simulation environment to accelerate the extensive cross-validation experiments and hyperparameter sweeps. In practical deployment, the local training and inference operations, based on our relatively lightweight ResNet-18 backbone, are intended to be executed directly on edge devices, potentially augmented with edge-optimized AI accelerators, rather than relying on workstation-class GPUs.

### 4.4. Baseline Methods

We compared the proposed method against the following baselines. Hyperparameters for each method were set to the values recommended in the respective original papers, with adjustments where necessary. For fair comparison, all methods except FedPos used the same ResNet-18 backbone, preprocessing pipeline, and data splits.

- No-Fed: A model trained solely on target domain data without federated learning. This represents performance when no prior knowledge from source domains is utilized, serving as a baseline for evaluating the benefit of knowledge transfer through federated learning.
- Centralized: A model trained on aggregated data from all source domains. This assumes an ideal scenario where all clients can share data, representing the performance upper bound without privacy constraints. In this study, it also serves as a reference for the upper bound for stability.
- FedAvg [12]: The standard federated learning algorithm. Each client independently updates the model on local data, and the server aggregates updates via weighted averaging proportional to data volume.
- FedProx [17] (μ=0.01): An extension of FedAvg that adds a proximal term to the loss function during local updates, limiting divergence from the global model. This regularization suppresses variability in model updates across clients under non-IID data conditions, stabilizing convergence.
- MOON [34] (μ=1.0, τ=0.5): A method integrating contrastive learning into federated learning. During local training, a contrastive loss is computed among the feature representations of the current local model, the previous local model, and the global model. This encourages the local model’s representation to align with the global model while diverging from the previous local model, promoting feature representation consistency across clients.
- Per-FedAvg [40]: A personalized federated learning method that applies the Model-Agnostic Meta-Learning (MAML) framework to FL. It optimizes the global model as an initialization suitable for fine-tuning on each client, designed with few-shot adaptation in mind.
- FedProc [35] (μ=0.1, τ=0.07): A federated learning method using prototype-based contrastive learning. Class-representative feature vectors (prototypes) are aggregated and shared by the server, aligning the feature space structure across clients while maintaining inter-class discriminability.
- FedSR [53]: A federated learning method targeting domain generalization. It constrains the $L^{2}$ norm of feature vectors to project them onto a unit hypersphere and minimizes conditional mutual information to suppress domain-specific information, aiming to improve generalization to unseen environments.
- FedPos [22]: A federated learning method designed for Wi-Fi localization. It explicitly separates the model into components shared across environments and environment-specific components, integrating a transfer learning mechanism. In this study, it serves as a reference for the upper bound for plasticity. Following the original paper, GhostNet [54] was used as the backbone.

### 4.5. Evaluation Metrics

Two metrics were used to evaluate localization performance.

Mean Localization Error (MLE) is the mean Euclidean distance between estimated and true positions:

(17) $$\mathrm{MLE}=\frac{1}{N}\sum_{i=1}^{N}{∥{\hat{p}}_{i}-p_{i}∥}_{2},$$

where *N* is the number of test samples, ${\hat{p}}_{i}$ is the estimated position for the *i*-th sample, and $p_{i}$ is the true position.

Root Mean Square Error (RMSE) is the square root of the mean squared error, which is more sensitive to large errors than MLE:

(18) $$\mathrm{RMSE}=\sqrt{\frac{1}{N}\sum_{i=1}^{N}{∥{\hat{p}}_{i}-p_{i}∥}_{2}^{2}}.$$

Although localization is formulated as RP classification in this study, predicted class labels are converted back to corresponding coordinates, so evaluation metrics are computed as distance errors in coordinate space (MLE and RMSE).

To ensure reproducibility, 5 independent runs were performed with random seeds {0,42,123,2025,3407} [55]. Due to computational constraints, the mean and standard deviation are reported only for methods for which evaluation across all seeds was completed. The statistical significance of differences between methods was assessed using paired *t*-tests (significance level α=0.05). Effect sizes were quantified using Cohen’s *d*, with |d|<0.2 indicating negligible effect, 0.2≤|d|<0.5 small, 0.5≤|d|<0.8 medium, and |d|≥0.8 large.

## 5. Results

### 5.1. Performance in Known Environments (Internal Validity)

Table 3 summarizes the localization performance on source clients. AdaFed-LDR achieved an MLE of 0.41±0.01 cm, representing an 88.2% reduction compared with FedAvg (3.47±0.23 cm). The difference relative to the next-best federated method, FedPos (1.31±0.20 cm), was statistically significant (p<0.001, Cohen’s d=4.31). The gap relative to the Centralized model (0.40±0.05 cm) was 0.01 cm.

The standard deviation of AdaFed-LDR across seeds (±0.01 cm) was substantially lower than that of FedPos (±0.20 cm). Under the classification-based formulation described in Section 3.2, a correct RP prediction yields near-zero localization error, while misclassification to an adjacent RP results in an error of at least 30 cm; an MLE of 0.41 cm thus corresponds to a misclassification rate of approximately 1.4%.

**Figure 4:**
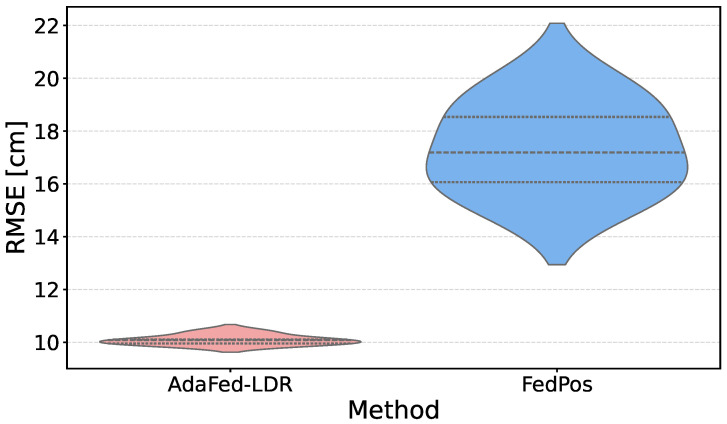
RMSE distribution in source domains comparing FedPos and AdaFed-LDR. Each violin represents 280 measurements (7 source clients × 8 LOOCV folds × 5 seeds), where the internal dashed lines indicate the third quartile, the median, and the first quartile from top to bottom. AdaFed-LDR exhibits a narrow distribution (interquartile range (IQR) ≈ 0.3 cm) compared to FedPos (IQR ≈ 3 cm), indicating approximately 10-fold reduction in variance across different random initializations.

Figure 4 visualizes the RMSE distributions in known environments. AdaFed-LDR’s distribution is concentrated around 10.09±0.19 cm, whereas FedPos shows a wider spread (17.36±1.72 cm). The IQR of AdaFed-LDR (approximately 0.3 cm) is roughly one-tenth that of FedPos (approximately 3 cm).

### 5.2. Adaptation Performance in Unknown Environments (External Validity)

Table 4 presents zero-shot and few-shot performance on unseen target environments.

In the Zero-Shot setting, AdaFed-LDR achieved an MLE of 218.23±2.80 cm. The difference from FedPos (257.64±14.04 cm) was statistically significant (p<0.01, Cohen’s d=2.78). The standard deviation of AdaFed-LDR (±2.80 cm) was approximately one-fifth that of FedPos (±14.04 cm).

In the 1-Shot setting, AdaFed-LDR achieved an MLE of 21.07±1.50 cm. FedPos achieved a lower MLE (13.93±2.76 cm, p<0.05, Cohen’s d=−2.04), with a standard deviation approximately 1.8 times that of AdaFed-LDR.

In the 5-Shot setting, FedPos achieved 5.12±1.03 cm compared to AdaFed-LDR’s 8.26±0.65 cm (p<0.01, Cohen’s d=−2.80). The standard deviation of FedPos (±1.03 cm) was approximately 1.6 times that of AdaFed-LDR (±0.65 cm).

**Figure 5:**
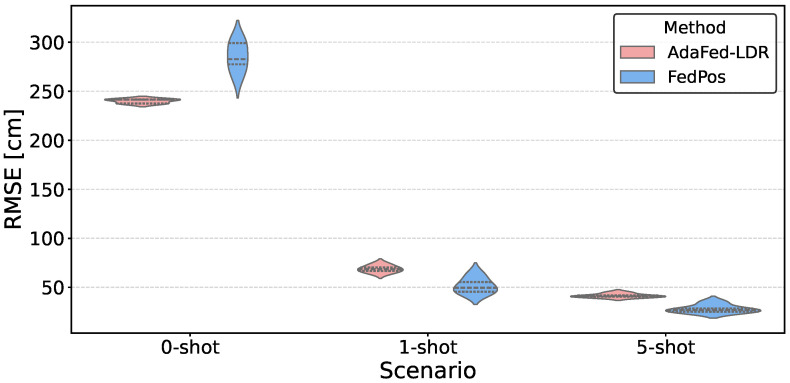
RMSE distributions across adaptation scenarios (zero-shot/1-shot/5-shot) in target domains. Each violin aggregates 40 measurements (8 LOOCV folds × 5 seeds), where the internal dashed lines indicate the third quartile, the median, and the first quartile from top to bottom. AdaFed-LDR exhibits narrower distributions across all scenarios; FedPos achieves lower medians in few-shot settings at the cost of higher variance.

Figure 5 visualizes RMSE distributions across adaptation scenarios. In the zero-shot setting, AdaFed-LDR shows a narrower distribution (RMSE ±2.19 cm) compared with FedPos (±15.03 cm). In the 1-shot setting, FedPos achieves a lower median with a wider spread (±7.96 cm vs. ±3.60 cm for AdaFed-LDR). The variance gap narrows progressively from zero-shot to 5-shot as more adaptation samples become available.

### 5.3. Ablation Study

An ablation study was conducted across 5 random seeds to quantify the contribution of each component. Four configurations were evaluated:

- Baseline (FedProx): Both proposed components removed.
- *w*/*o* LDR: Adaptive aggregation only.
- *w*/*o* Aggregation: LDR only.
- Full: Both components applied (AdaFed-LDR).

#### 5.3.1. Performance in Known Environments

Table 5 presents the ablation results for source domains. The Full configuration achieved an MLE of 0.41±0.01 cm. Removing adaptive aggregation increased the MLE to 1.74±0.05 cm, and removing LDR increased it to 1.92±0.22 cm. The baseline (FedProx) yielded 2.53±0.17 cm. The standard deviation when removing LDR (±0.22 cm) was larger than when removing adaptive aggregation (±0.05 cm) or using the Full configuration (±0.01 cm).

**Figure 6:**
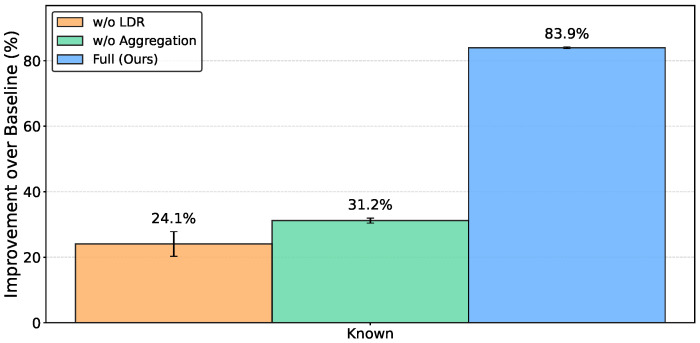
Relative improvement over FedProx baseline in known environments, averaged across 5 random seeds. The Full configuration achieves 83.9% improvement, yielding the highest accuracy and the lowest variance compared to the individual components. Error bars represent standard deviations across seeds.

Figure 6 shows the relative improvement of each configuration over the FedProx baseline. Compared to the individual components (24.1% for *w*/*o* LDR and 31.2% for *w*/*o* Aggregation), the Full configuration achieved the highest overall improvement of 83.9%. The Full configuration also exhibited the smallest variance across random seeds, suggesting that the two mechanisms effectively complement each other to stabilize learning.

#### 5.3.2. Performance in Unknown Environments

Table 6 presents the ablation results for target domains across zero-shot, 1-shot, and 5-shot settings.

In the Zero-Shot setting, the *w*/*o* LDR configuration (215.85±2.82 cm) marginally outperformed the Full configuration (218.23±2.80 cm). In the 1-Shot setting, the Full configuration achieved 21.07±1.50 cm, compared with 23.45±2.83 cm for *w*/*o* Aggregation and 23.49±1.74 cm for *w*/*o* LDR. In the 5-Shot setting, the *w*/*o* Aggregation configuration (8.12±0.82 cm) marginally outperformed the Full configuration (8.26±0.65 cm), while *w*/*o* LDR showed the highest variance (±1.80 cm, approximately 2.8 times that of the Full configuration).

**Figure 7:**
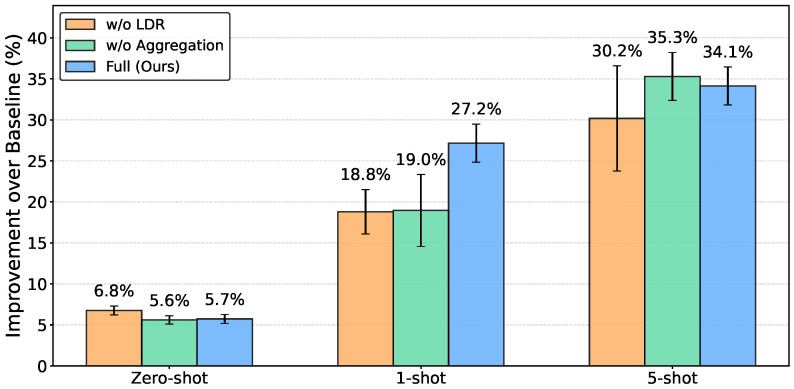
Relative improvement over FedProx baseline in unknown environments across adaptation scenarios, averaged across 5 random seeds. The Full configuration achieves 5.7% improvement in zero-shot, 27.2% in 1-shot, and 34.1% in 5-shot settings. Error bars represent standard deviations across seeds.

Figure 7 shows the relative improvement over FedProx in the target domains. The Full configuration achieved 5.7% improvement in zero-shot, 27.2% in 1-shot, and 34.1% in 5-shot settings, with consistently narrow error bars. The relatively small and variable improvements in the zero-shot setting across all configurations, combined with larger error bars, indicate that the effects of each component are more pronounced when adaptation data are available.

## 6. Discussion

### 6.1. High Precision in Source Domains

Under the classification-based formulation described in Section 3.2, a correct RP prediction yields near-zero localization error, whereas misclassification to an adjacent RP incurs an error of at least 30 cm. The MLE of 0.41 cm achieved by AdaFed-LDR in source domains corresponds to a misclassification rate of approximately 1.4%, suggesting that the global backbone has learned discriminative representations across all source environments. In addition, even when the task is formulated as a coordinate regression, AdaFed-LDR achieves a Mean Localization Error (MLE) of 12.27 cm, compared to 44.33 cm for FedAvg (Appendix A). This result further indicates that the global backbone learned highly discriminative and robust spatial representations across diverse environments.

The gap of 0.01 cm relative to the Centralized model (Table 3) suggests that federated training can maintain localization precision comparable to centralized approaches while preserving data locality, at least under identical hardware configurations.

However, it should be noted that these results are contingent upon a strictly static environment where the receiver was placed directly on the floor in an empty room, free from the interference of moving objects or people, as detailed in Section 4.1.2. Whether this result generalizes to heterogeneous hardware settings remains an open question requiring further empirical investigation.

### 6.2. Stability–Plasticity Trade-Off in Practice

Table 3 and Table 4 indicate that FedPos and AdaFed-LDR occupy distinct operating points on the stability–plasticity spectrum. FedPos demonstrates superior few-shot adaptation at the cost of source-domain precision (MLE 1.31 cm, approximately 3.3× that of the Centralized model), whereas AdaFed-LDR maintains near-Centralized source-domain precision while showing comparatively lower few-shot adaptation accuracy. The performance gap between the two methods narrows as the amount of adaptation data increases, suggesting that additional calibration samples can progressively compensate for AdaFed-LDR’s lower inherent plasticity.

The appropriate choice between these frameworks depends on the constraints of the target application. For systems requiring sustained, high-precision localization within previously calibrated environments—such as asset tracking in automated warehouses—AdaFed-LDR is a suitable candidate. For scenarios where rapid adaptation to uncalibrated environments is prioritized, FedPos offers a more effective approach.

### 6.3. Comparison Between Federated and Centralized Learning

As shown in Table 4, AdaFed-LDR outperformed the Centralized model in target-domain adaptation while achieving equivalent precision in source domains. In the 1-shot setting, AdaFed-LDR achieved approximately one-third the error of the Centralized model; a similar trend was observed in the 5-shot setting.

Two hypotheses may explain this observation. First, independent local optimization in federated training may promote the retention of environment-specific signal characteristics within intermediate feature representations, whereas joint optimization across diverse domains in centralized training may attenuate these characteristics through gradient averaging. Second, the adaptive aggregation mechanism, which down-weights updates from clients exhibiting irregular covariance shifts, may facilitate the retention of transferable generic features in the global model. Neither hypothesis was directly verified in this study; rigorous analysis of feature-space distributions and gradient trajectories is required to substantiate these interpretations.

### 6.4. Performance of Per-FedAvg

Per-FedAvg demonstrated substantially higher source-domain MLE than the other federated baselines (Table 3), with few-shot adaptation performance similarly limited (Table 4).

Per-FedAvg applies the Model-Agnostic Meta-Learning (MAML) framework, which assumes a relatively homogeneous class structure across tasks. In the present experimental setting, each environment comprises a varying number of RPs with distinct spatial layouts, and class labels do not correspond across domains. This structural heterogeneity likely destabilized the inner-loop gradient computation required by MAML. A systematic hyperparameter search for inner-loop optimization may improve performance; however, such an investigation is beyond the scope of the present study.

### 6.5. Observations on Contrastive Learning Methods

Contrastive learning approaches (MOON and FedProc) did not outperform FedAvg in the adaptation setting (Table 4).

A plausible contributing factor is the physical continuity inherent in CSI data. Because radio channel responses vary continuously with spatial displacement, adjacent RPs produce highly correlated CSI patterns. Contrastive objectives enforce feature separation between different classes (negative pairs); applying this constraint to physically adjacent RPs may disrupt the spatial continuity of the learned feature manifold, potentially degrading adaptation performance. This hypothesis warrants quantitative verification via topological analysis of the feature space, for example through Uniform Manifold Approximation and Projection (UMAP), which is reserved for future work.

### 6.6. Reproducibility Across Initializations

AdaFed-LDR demonstrated substantially lower performance variance compared to baseline methods across different random initializations, as shown in Table 3 and Table 4.

Ablation results (Table 5) indicate that removing LDR markedly increased source-domain standard deviation. One possible interpretation is that constraining the updates of shallow layers via LDR restricts the accessible parameter space during training, directing convergence toward structurally similar local minima regardless of initialization. Verification of this interpretation through loss-landscape geometry analysis is a direction for future work. From a practical standpoint, reduced variance contributes to predictable and stable deployment without requiring multiple training runs.

### 6.7. Limitations of Zero-Shot Performance

Both evaluated methods yielded substantial zero-shot errors (Table 4). This reflects a fundamental constraint of CSI sensing: multipath characteristics are dominated by environment-specific factors such as room geometry and material permittivity, which cannot be inferred from source-domain data alone.

While an MLE exceeding 200 cm is insufficient for precise localization, the 1-shot adaptation result (Table 4) demonstrates that a minimal calibration sample of approximately 0.31 s per RP can substantially restore operational accuracy. This characteristic suggests a practical deployment strategy for large-scale indoor positioning systems, where exhaustive prior calibration is often impractical.

### 6.8. Methodological Limitations

#### 6.8.1. Temporal Variation

The dataset was collected in single, contiguous sessions between April and October 2025. However, CSI is known to be sensitive to temporal environmental changes. For example, it has been reported that variations in indoor temperature and humidity, as well as the movement of furniture and other objects, can significantly alter CSI distributions and potentially affect the robustness of downstream inference [56]. Therefore, the resilience of AdaFed-LDR against long-term temporal drift was not evaluated in the present study. Developing an online continual learning mechanism to accommodate temporal domain shifts is an important direction for future work.

#### 6.8.2. Hardware Homogeneity

The experiments relied exclusively on uniform hardware: Raspberry Pi 4 Model B and ASUS RT-AC86U. Although real-world federated scenarios may involve hardware heterogeneity, this uniform setup was a deliberate experimental design choice to isolate environmental non-IIDness, caused by room geometry and multipath interference, from hardware-induced non-IIDness, such as device-specific amplitude-scaling biases. If heterogeneous hardware had been used, these two fundamentally distinct sources of domain shift would have been confounded, making it more difficult to evaluate AdaFed-LDR’s environmental adaptation capability. In realistic federated scenarios, client devices may exhibit hardware-induced biases in amplitude scaling, which could cause the adaptive aggregation module to misinterpret such biases as unstable learning and penalize the affected clients. However, addressing these hardware differences is an orthogonal challenge; developing hardware-agnostic calibration layers at the preprocessing stage is a prerequisite for practical deployment in heterogeneous device environments.

#### 6.8.3. Communication Overhead

Transmitting feature covariance matrices introduces additional communication overhead. Because federated learning relies on periodic, bursty model updates rather than continuous data streaming, this overhead is more appropriately evaluated in terms of data volume per round rather than continuous bit rate. Specifically, for the 512-dimensional output of ResNet-18, a covariance matrix requires approximately 0.5 MB per client per round. In standard federated learning, only the backbone parameters are transmitted, resulting in approximately 10 MB of communication per round. In contrast, our proposed method additionally transmits the feature covariance matrix, resulting in an approximately 5% increase in transmitted data volume.

Although this overhead is modest under typical network conditions, it may be restrictive in bandwidth-constrained deployments. Future work should explore low-rank approximations or matrix quantization to reduce transmission cost.

#### 6.8.4. Frame Rate and Window Size Selection

Our system currently uses a transmission rate of 800 Hz and a sliding window of 250 packets, corresponding to approximately 0.31 s of temporal information per sample. The transmission rate and the sliding window size are hyperparameters of the proposed system. Their selection should depend on the requirements of the target deployment scenario and the constraints of the available hardware. Accordingly, empirically optimizing these temporal parameters for specific application settings remains an important direction for future research.

#### 6.8.5. Covariance-Based Reliability Estimation

Our adaptive aggregation mechanism empirically assumes that exceptionally large or non-uniform covariance shifts indicate unreliable updates. However, this assumption has not been validated under mathematically controlled perturbations. For instance, if a client experiences a sudden but legitimate structural reconfiguration (e.g., a permanent layout modification, as discussed in Section 6.8.1), the resulting large covariance shift might be misclassified as transient noise. Consequently, the server would inappropriately down-weight the client, delaying the global model’s adaptation to the valid environmental change. Furthermore, while the current framework utilizes the geometric median as a heuristic robust aggregator, its integration with established robust statistical estimation frameworks has not yet been explored. In real-world Wi-Fi sensing, dynamic multipath interference often induces non-stationary, heavy-tailed measurement noise that violates Gaussian assumptions. To provide a principled handling of such non-Gaussian uncertainty and accurately distinguish between genuine domain shifts and heavy-tailed noise, future work should explore connections to robust Bayesian filtering and heavy-tailed modeling. For example, incorporating advanced frameworks such as robust Kalman filtering based on Normal–Bernoulli distributions [57] could provide a more principled framework for handling non-Gaussian uncertainty in client reliability estimation. Integrating these robust statistical frameworks with online continual learning mechanisms to dynamically handle temporal environmental variations (Section 6.8.1) is expected to be an important direction for future research.

### 6.9. Privacy Considerations

While AdaFed-LDR prevents the direct transmission of raw CSI data and location labels, the exchange of model parameters and feature covariance matrices, as in typical federated learning, could introduce a secondary privacy vulnerability. Specifically, feature covariance matrices could potentially be exploited to infer statistical properties of the local environment [58,59].

Differential Privacy (DP) [60] could theoretically mitigate this risk by injecting calibrated noise into the covariance matrices before transmission. However, additive noise alters the covariance structure, and the extent to which DP constraints degrade the accuracy of the adaptive aggregation mechanism remains to be quantified [61]. Investigating this trade-off between privacy protection and aggregation quality is an important direction for future work.

## 7. Conclusions

This paper proposed AdaFed-LDR to address the stability–plasticity trade-off in federated CSI-based indoor localization. AdaFed-LDR combines server-side adaptive aggregation based on feature covariance changes with client-side Layerwise Dynamics Regularization (LDR), which imposes stronger constraints on shallow layers to preserve generic features shared across environments and weaker constraints on deeper layers to allow environment-specific adaptation.

Evaluation across 8 indoor environments with 5 random seeds confirmed that AdaFed-LDR achieves source-domain precision comparable to centralized learning while outperforming existing federated methods in domain generalization to unseen environments. In few-shot adaptation, AdaFed-LDR achieves lower error than centralized learning with only one sample per reference point, suggesting that a minimal calibration budget is sufficient for practical deployment. Although FedPos achieves lower adaptation error in few-shot settings, AdaFed-LDR exhibits substantially smaller performance variance across seeds, indicating higher reproducibility. Ablation experiments confirmed that combining the two components yields the highest overall improvement, supporting the design rationale of addressing layer-specific roles in both aggregation and regularization.

This study has several limitations. First, the dataset was collected in static environments over a fixed period, and robustness to long-term temporal drift caused by changes in temperature, humidity, or furniture placement has not been evaluated. Second, all experiments used uniform hardware, and the behavior of the adaptive aggregation module under hardware-induced amplitude and phase biases remains to be examined. Third, the evaluation covered 8 environments within a single institution, and validation across a larger and more architecturally diverse set of buildings is necessary to establish broader generalizability.

Future directions include integrating established robust statistical estimation frameworks with online continual learning mechanisms to dynamically handle temporal domain shifts and non-Gaussian measurement noise. Additionally, we plan to explore hardware-agnostic calibration strategies, communication-efficient matrix approximations, and large-scale cross-building evaluation. Integration with differential privacy techniques to protect the transmitted covariance matrices is also an important direction. We hope this study contributes to the broader understanding of how layer-specific regularization can mitigate the stability–plasticity trade-off in federated learning for CSI-based localization.

## Figures and Tables

**Table 1:** Data collection summary across 8 heterogeneous environments. Total dataset comprises 111,888 preprocessed tensors from 434 reference points collected during April–October 2025 using consistent hardware (Raspberry Pi 4B + ASUS RT-AC86U).

ID	Environment Description	RPs	Samples (Tensors)
A	Server Room (Info. Bldg. 1, 4F)	11	2873
B	Residential Apartment	27	7220
C	Common Area-S (Info. Bldg. 2, 2F)	44	11,676
D	Common Area (Info. Bldg. 1, 1F)	50	13,795
E	Common Area (Info. Bldg. 2, 8F)	61	15,124
F	Common Area (Info. Bldg. 2, 4F)	68	17,539
G	Common Area-L (Info. Bldg. 2, 2F)	82	21,013
H	Common Area (Eng. Bldg. 6, 1F)	91	22,648
Total	–	434	111,888

RPs: Unique ground-truth reference points at 30 cm spacing. Tensors: Image samples (224×224×3) after sliding window preprocessing.

**Table 2:** Hyperparameter settings for AdaFed-LDR. Federated learning and adaptation phase parameters follow values reported in FedAvg and FedPos. AdaFed-LDR-specific parameters follow Section 4.3.4.

Category	Parameter	Value
Federated Learning	Optimizer	AdamW
	Learning rate	0.001
	Weight decay	0.01
	Batch size	512
	Communication rounds	300
	Local epochs per round	1
	Scheduler	StepLR (step 50, γ=0.5)
Few-Shot Adaptation	Learning rate	0.0001
	Epochs	80
AdaFed-LDR	Aggregation temperature *T*	1.0
	Confidence coefficients $\lambda_{1},\lambda_{2},\lambda_{3}$	0.5, 1.0, 0.1
	LDR regularization coefficient $\lambda_{\mathrm{LDR}}$	0.001
	Layer-specific reg. coefficients $\eta_{S},\eta_{M},\eta_{D}$	1.0, 0.5, 0.1
	Layer importance weights $\alpha_{S},\alpha_{M},\alpha_{D}$	0.25, 0.35, 0.40
	EMA coefficient β	0.9
	Proximal reg. coefficient μ	0.01

**Table 3:** Localization performance on source domains (known environments). Mean Localization Error (MLE) and Root Mean Square Error (RMSE) averaged over 7 source clients using leave-one-out cross-validation (LOOCV) with 5 random seeds. Paired *t*-test assesses statistical significance between FedPos and AdaFed-LDR.

Method	MLE (cm)	RMSE (cm)
Centralized	0.40±0.05	9.93±0.63
FedAvg	3.47±0.23	30.92±1.00
FedProx	2.53±0.17	25.39±0.87
MOON	3.67±0.12	30.94±0.71
Per-FedAvg	233.44±8.70	263.17±9.82
FedProc	3.95±0.34	32.48±1.59
FedSR	3.99±0.22	33.22±1.37
FedPos	1.31 ± 0.20	17.36 ± 1.72
AdaFed-LDR (Ours)	0.41 ± 0.01 ***	10.09 ± 0.19 ***

**Bold**: best federated method; Underline: second-best. *** p<0.001 (paired *t*-test, N=40: 8 folds × 5 seeds, Cohen’s d=4.31). Values: mean ± std across seeds.

**Table 4:** Few-shot adaptation performance on target domains (unknown environments). Zero-shot applies the pretrained global model without adaptation. *K*-shot fine-tunes using the chronologically first *K* samples per RP (80 epochs, lr = 0.0001). Results averaged over 8 target clients (LOOCV) with 5 seeds.

Method	Zero-Shot (K=0)		1-Shot (K=1)		5-Shot (K=5)	
	MLE	RMSE	MLE	RMSE	MLE	RMSE
No-Fed	-	-	57.59±2.76	113.79±3.42	12.25±0.59	51.69±1.69
Centralized	233.00±4.71	259.38±4.09	64.07±5.13	128.90±6.03	24.14±3.17	72.34±5.56
FedAvg	238.60±2.67	264.87±2.87	29.91±3.13	77.96±3.01	11.87±1.63	44.79±2.92
FedProx	231.50±3.06	256.72±2.47	28.93±1.41	77.04±5.49	12.55±2.36	45.30±2.25
MOON	237.83±2.92	264.07±3.44	32.05±2.72	80.50±4.71	11.63±2.36	43.99±5.76
Per-FedAvg	230.51 ± 9.88	252.84 ± 8.67	53.96±6.46	109.55±8.62	9.91±0.93	44.59±3.30
FedProc	238.87±3.36	264.82±3.60	31.76±2.45	81.50±4.26	12.73±2.43	47.33±4.91
FedSR	239.37±2.68	264.81±2.20	34.89±3.01	86.21±2.31	16.37±2.56	53.50±5.53
FedPos	257.64±14.04	284.89±15.03	13.93 ± 2.76 *	51.60 ± 7.96 *	5.12 ± 1.03 **	27.98 ± 4.15 **
AdaFed-LDR (Ours)	218.23 ± 2.80 **	239.89 ± 2.19 **	21.07 ± 1.50	68.72 ± 3.60	8.26 ± 0.65	41.45 ± 1.98

**Bold**: best; Underline: second-best. All values in cm. * p<0.05, ** p<0.01 (paired *t*-test, N=40, Cohen’s |d|>1.8). Values: mean ± std across 5 seeds.

**Table 5:** Ablation study results for known environments (source domains), averaged across 5 random seeds and 8 LOOCV folds (40 measurements per configuration). Baseline uses FedProx (μ=0.01) without proposed components.

Configuration	MLE (cm)	RMSE (cm)
Baseline (FedProx)	2.53 ± 0.17	25.39 ± 0.86
*w*/*o* LDR	1.92 ± 0.22	23.29 ± 1.23
*w*/*o* Aggregation	1.74 ± 0.05	23.12 ± 0.18
Full (Ours)	**0.41 ± 0.01**	**10.09 ± 0.19**

**Bold**: best; Underline: second-best. All values in cm. Values: mean ± std across 5 seeds. Each mean aggregates 8 LOOCV folds.

**Table 6:** Ablation study results for unknown environments (target domains), averaged across 5 random seeds and 8 LOOCV folds (40 measurements per configuration). Baseline uses FedProx (μ=0.01) without proposed components.

Configuration	Zero-Shot		1-Shot		5-Shot	
	MLE	RMSE	MLE	RMSE	MLE	RMSE
Baseline (FedProx)	231.50±3.06	256.72±2.47	28.93±1.41	77.04±5.49	12.55±2.36	45.30±2.25
*w*/*o* LDR	**215.85 ± 2.82**	**237.83 ± 2.65**	23.49±1.74	74.33 ± 5.29	8.76±1.80	42.88±6.28
*w*/*o* Aggregation	218.51±2.68	239.69 ± 2.45	23.45 ± 2.83	74.76±5.65	**8.12 ± 0.82**	**40.02 ± 4.23**
Full (Ours)	218.23 ± 2.80	239.89±2.19	**21.07 ± 1.50**	**68.72 ± 3.60**	8.26 ± 0.65	41.45 ± 1.98

**Bold**: best; Underline: second-best. All values in cm. Values: mean ± std across 5 seeds. Each mean aggregates 8 LOOCV folds.

**Figure A1:**
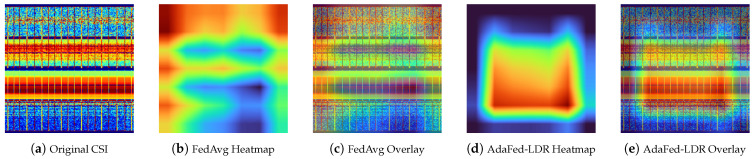
Grad-CAM++ attention visualization comparing FedAvg and AdaFed-LDR. (**a**) Original preprocessed CSI input. (**b**,**c**) FedAvg exhibits distributed attention across the frequency range, including peripheral bands. (**d**,**e**) AdaFed-LDR shows spatially concentrated attention on the central subcarrier bands. Red indicates high attention; blue indicates low attention.

## Data Availability

The data presented in this study are not publicly available due to privacy restrictions.

## Appendix A. Performance Evaluation on Regression Task

This appendix evaluates the performance of AdaFed-LDR when localization is formulated as a regression problem. While the main body of this paper employs a classification-based approach using the Adaptive Grid Converter, the regression model directly predicts continuous (x,y) coordinates. For the regression task, the classification head was replaced with a linear layer outputting 2-dimensional coordinates, optimized via Mean Squared Error (MSE) loss (seed: 42).

FedPos, FedProc, and FedSR were excluded from this evaluation. These methods rely on mechanisms designed for classification tasks—such as prototype matching and class-specific distribution alignment—and are not directly applicable to continuous coordinate regression.

### Appendix A.1. Source Domain Performance

Table A1 presents the regression performance in source domains. Centralized learning achieved an MLE of 3.06 cm. Among the federated methods, AdaFed-LDR achieved the lowest error with an MLE of 12.27 cm, outperforming FedAvg (44.33 cm) and FedProx (49.55 cm).

The classification-based approach (Table 3) achieved an MLE of 0.41 cm for AdaFed-LDR, approximately 30 times lower than the regression formulation (12.27 cm). This difference can be attributed to the fact that in classification over discrete RPs, the error is nearly zero when classification is correct, whereas in regression, prediction errors in continuous coordinates accumulate.

**Table A1:** Regression formulation performance on source domains. Model outputs continuous (x,y) coordinates optimized via MSE loss. The classification-based approach (Table 3) achieves approximately 30× lower MLE.

Method	MLE (cm)	RMSE (cm)
Centralized	3.06	10.00
FedAvg	44.33	61.43
FedProx	49.55	67.11
MOON	45.97	64.18
Per-FedAvg	181.42	196.65
AdaFed-LDR (Ours)	**12.27**	**20.32**

FedPos, FedProc, FedSR excluded (classification-specific mechanisms). **Bold**: best federated; Underline: second-best.

### Appendix A.2. Target Domain Performance

Table A2 presents the zero-shot and few-shot performance in target domains.

In the 1-shot setting, AdaFed-LDR achieved an MLE of 45.33 cm, lower than FedProx (49.46 cm) and FedAvg (52.73 cm). In the 5-shot setting, AdaFed-LDR also achieved the lowest error at 27.12 cm. In the zero-shot setting, Per-FedAvg achieved the lowest error at 182.55 cm, while AdaFed-LDR achieved 191.75 cm.

Compared to the classification-based approach (Table 4), the absolute errors in the regression formulation are higher. For example, in the 5-shot setting, the classification approach achieved 8.26 cm while the regression formulation yielded 27.12 cm. However, the relative advantage of AdaFed-LDR over other federated methods is maintained in the regression formulation as well.

**Table A2:** Regression formulation performance on target domains. AdaFed-LDR maintains its relative advantage over baselines in few-shot scenarios despite higher absolute errors compared to the classification approach (Table 4).

Method	Zero-Shot (K=0)		1-Shot (K=1)		5-Shot (K=5)	
	MLE	RMSE	MLE	RMSE	MLE	RMSE
No-Fed	–	–	78.07	95.72	35.02	51.93
Centralized	202.32	225.40	86.76	114.52	59.56	84.03
FedAvg	191.64	212.69	52.73	76.36	34.60	54.61
FedProx	192.35	211.78	49.46	69.82	31.15	47.59
MOON	192.20	213.33	51.61	71.94	33.19	49.99
Per-FedAvg	**182.55**	**192.90**	77.33	91.02	44.36	58.19
AdaFed-LDR (Ours)	191.75	205.40	**45.33**	**63.98**	**27.12**	**43.03**

All values in cm. **Bold**: best; Underline: second-best.

## Appendix B. Visualization of Model Attention via Grad-CAM++

To qualitatively evaluate which regions of the CSI data contribute to model predictions, we applied Grad-CAM++ to the final convolutional layer (layer4) of both FedAvg and AdaFed-LDR. Figure A1 presents attention maps overlaid on the input CSI amplitude images (vertical axis: subcarrier index; horizontal axis: time).

The FedAvg attention map (Figure A1b,c) is distributed broadly across both frequency and time dimensions. Activation is observed in the peripheral frequency bands (top and bottom regions of the image), which tend to have lower signal-to-noise ratios due to the guard band characteristics of OFDM systems. This distributed pattern suggests that FedAvg may not sufficiently distinguish between discriminative signal features and environment-specific noise.

The AdaFed-LDR attention map (Figure A1d,e) is concentrated in the central subcarrier bands. This region corresponds to areas in the original CSI image where structured temporal patterns are observed. Lower attention weights (blue regions) are assigned to the peripheral frequency bands, suggesting suppression of noise-prone components.

This difference in attention patterns is potentially consistent with the cross-seed stability of AdaFed-LDR shown in Table 3. The concentration of attention on the central subcarrier bands suggests that features are extracted from physically consistent signal regions rather than from environment-specific noise.

However, Grad-CAM++ reflects gradient flow patterns and does not directly indicate causal relationships between input features and predictions. A quantitative assessment would require statistical analysis of attention distributions across the entire dataset and multiple network layers.
